# Alterations in static and dynamic regional homogeneity in mesial temporal lobe epilepsy with and without initial precipitating injury

**DOI:** 10.3389/fnins.2023.1226077

**Published:** 2023-08-01

**Authors:** Xinyue Mao, Xiaonan Zhang, Chengru Song, Keran Ma, Kefan Wang, Xin Wang, Yajun Lian, Yong Zhang, Shaoqiang Han, Jingliang Cheng, Yan Zhang

**Affiliations:** ^1^Department of Magnetic Resonance Imaging, The First Affiliated Hospital of Zhengzhou University, Zhengzhou, China; ^2^Key Laboratory for Functional Magnetic Resonance Imaging and Molecular Imaging of Henan Province, Zhengzhou, China; ^3^Engineering Technology Research Center for Detection and Application of Brain Function of Henan Province, Zhengzhou, China; ^4^Engineering Research Center of Medical Imaging Intelligent Diagnosis and Treatment of Henan Province, Zhengzhou, China; ^5^Key Laboratory of Magnetic Resonance and Brain Function of Henan Province, Zhengzhou, China; ^6^Key Laboratory of Brain Function and Cognitive Magnetic Resonance Imaging of Zhengzhou, Zhengzhou, China,; ^7^Key Laboratory of Imaging Intelligence Research Medicine of Henan Province, Zhengzhou, China; ^8^Department of Neurology, The First Affiliated Hospital of Zhengzhou University, Zhengzhou, China

**Keywords:** mesial temporal lobe epilepsy, initial precipitating injury, resting-state functional MRI, regional homogeneity, dynamic, cognitive impairment

## Abstract

**Objectives:**

Initial precipitating injury (IPI) such as febrile convulsion and intracranial infection will increase the susceptibility to epilepsy. It is still unknown if the functional deficits differ between mesial temporal lobe epilepsy with IPI (mTLE-IPI) and without IPI (mTLE-NO).

**Methods:**

We recruited 25 mTLE-IPI patients, 35 mTLE-NO patients and 33 healthy controls (HC). Static regional homogeneity (sReHo) and dynamic regional homogeneity (dReHo) were then adopted to estimate the alterations of local neuronal activity. One-way analysis of variance was used to analyze the differences between the three groups in sReHo and dReHo. Then the results were utilized as masks for further between-group comparisons. Besides, correlation analyses were carried out to detect the potential relationships between abnormal regional homogeneity indicators and clinical characteristics.

**Results:**

When compared with HC, the bilateral thalamus and the visual cortex in mTLE-IPI patients showed an increase in both sReHo and variability of dReHo. Besides, mTLE-IPI patients exhibited decreased sReHo in the right cerebellum crus1/crus2, inferior parietal lobule and temporal neocortex. mTLE-NO patients showed decreased sReHo and variability of dReHo in the bilateral temporal neocortex compared with HC. Increased sReHo and variability of dReHo were found in the bilateral visual cortex when mTLE-IPI patients was compared with mTLE-NO patients, as well as increased variability of dReHo in the left thalamus and decreased sReHo in the right dorsolateral prefrontal cortex. Additionally, we discovered a negative correlation between the national hospital seizure severity scale testing score and sReHo in the right cerebellum crus1 in mTLE-IPI patients.

**Conclusion:**

According to the aforementioned findings, both mTLE-IPI and mTLE-NO patients had significant anomalies in local neuronal activity, although the functional deficits were much severer in mTLE-IPI patients. The use of sReHo and dReHo may provide a novel insight into the impact of the presence of IPI on the development of mTLE.

## Introduction

Mesial temporal lobe epilepsy (mTLE), characterized by transient aberrant electrical activity originating in the mesial temporal lobe structures (Morgan et al., 2020), is the most common focal drug-resistant epilepsy (Li et al., 2022). The hippocampus is particularly vulnerable to an intracranial infection or febrile convulsion (what we refer to as the initial precipitating injury, or IPI) in childhood (Bien et al., 2007; Polat et al., 2010; Janz et al., 2018), and this vulnerability often leads to hippocampal sclerosis, which is the most common cause of mTLE.

Whether there is IPI may be one of the factors leading to the alterations in brain’s structure or function of mTLE patients. It had been reported (Keller et al., 2002) that mTLE patients with a history of childhood febrile convulsions had smaller hippocampal and amygdala volumes in the ipsilateral side of the epileptic foci than those without such a history. Another study (Free et al., 1996) indicated that over half of the subjects with a history of encephalitis or meningitis experienced bilateral hippocampal volume loss. However, all of the current researches focused on alterations in brain structure, leaving the study of brain function in mTLE-IPI patients in the dark. Recently, a notion that there was a separation of cognitive problems and epileptogenesis was proposed (Patterson et al., 2014), which implied that certain kids with a history of febrile convulsions might have memory deficits that preceded the onset of mTLE. So it becomes sense to think that IPI has a high potential for promoting cognitive deterioration.

Resting-state functional magnetic resonance imaging (rs-fMRI) is a powerful tool for measuring the changes of blood oxygen level-dependent (BOLD) signals spontaneously produced by brain (Song and Jiang, 2012). One of the most well-liked rs-fMRI analysis methods, static regional homogeneity (sReHo), is a voxel-based approach that reflects the consistency of neural activity in a given voxel with its adjacent voxels (Zang et al., 2004). Contrary to the functional connection (FC), which depicts the inter-nodal connection, sReHo assesses intra-nodal activity (Hu et al., 2017). An abnormal sReHo indicates poor synchronization of local neural activity. It is well known that the sReHo of numerous brain regions, such as sensorimotor cortex, frontoparietal cortex and DMN, has been demonstrated to be disrupted in mTLE (Zeng et al., 2013; Zhao et al., 2020), which has a close relationship with the cognitive impairment of patients.

Considering the dynamic property of brain activity (Calhoun et al., 2014), recent investigations (Jiang et al., 2021; Liang et al., 2021) had explored the collaboration of brain regions by measuring the time-varying covariance of their neural signals during resting-state, among which dynamic regional homogeneity (dReHo) is a relatively common approach. An rs-fMRI study (Song et al., 2022) reported decreased variability of dReHo in the temporal lobe neocortex ipsilateral to epileptic foci in mTLE. Another study reported (Xue et al., 2022) that patients with major depressive disorder had decreased variability of dReHo in some brain regions associated with emotional and cognitive regulation, including the fusiform gyri, the right temporal pole and hippocampus. These suggest that plenty of diseases are accompanied by impaired regional temporal synchronization of spontaneous brain activity among certain voxels.

Based on the information presented above, regional homogeneity (ReHo) can be used to quantify activity coordination among voxels in a region. Since epilepsy is characterized by abnormal spontaneous brain activity, we predicted that the local neuronal activity of the epileptic foci and the brain regions affected by abnormal discharge transmission is impaired to some extent. From this point of view, the sReHo and dReHo were employed to characterize and compare the differences of neuronal activity among the three subgroups in this study. We hypothesized that (1) mTLE-IPI patients had more extensive alterations of sReHo or dReHo than mTLE-NO patients and (2) sReHo or dReHo in certain brain regions might be associated with some clinical characteristics of mTLE patients.

## Materials and methods

### Participants

BOLD-fMRI data were gathered in 60 unilateral mTLE cases (27 left- and 33 right-side) in The First Affiliated Hospital of Zhengzhou University between April 2019 and July 2022. At the same time, we recruited 33 age- and sex-matched healthy controls (HC) from the local communities, who also underwent BOLD-fMRI scans.

The diagnosis of mTLE was based on clinical symptom, neurologic examination, electroencephalography (EEG) and MRI findings, and was confirmed by neurologists from the department of neurology, The First Affiliated Hospital of Zhengzhou University. Patients with mTLE who met the following criteria were included: (1) MRI revealed that there were no obvious structural abnormalities other than unilateral hippocampal sclerosis, (2) all right-handed, (3) mini-mental state examination score > 24, and (4) ≥14 years old. Exclusion criteria for patients were as follows: (1) patients with other brain structural abnormalities except for unilateral hippocampal sclerosis, other psychiatric disorders, severe systemic diseases or trauma, (2) left-handed, and (3) had a long history of alcohol or drug abuse. According to whether there was intracranial infection or febrile convulsion in childhood, the patients were divided into two groups: mTLE with IPI (mTLE-IPI, *n* = 25) group and mTLE without IPI (mTLE-NO, *n* = 35) group. The mTLE-IPI group included 13 patients with a history of febrile convulsion and 12 patients with a history of intracranial infection (1 meningitis, 11 encephalitis).

The inclusion criteria for HC were as follows: (1) routine conventional MRI findings were normal, (2) had not taken any psychotropic tablets, and (3) all right-handed. The exclusion criteria: people with any family history of epilepsy or other psychiatric disorders.

Basic demographic data, including gender and age, was collected from all participants. The mTLE patients’ seizure-related characteristics, such as epilepsy duration, seizure severity, and the number of concurrent antiseizure medications (ASMs) were noted. Seizure severity was determined by the national hospital seizure severity scale (NHS3) testing score.

This study was approved by the Research Ethics Committee at The First Affiliated Hospital of Zhengzhou University (No.2019-KY-232). According to the Declaration of Helsinki, all participants were fully informed of the purpose of this study and provided written informed consent.

### MRI data acquisition

Resting-state fMRI data were acquired using a 3.0 T Magnetom Prisma MRI scanner (Siemens Healthcare, Erlangen, Germany), equipped with a 64-channel head coil. All participants were instructed to lie on their backs with their eyes closed, but to stay awake and to unconsciously relax. In order to reduce noise disturbance and minimize head motion, each participant was provided with a pair of sponge paddings. The scanning parameters were as follows: time of repetition (TR) =1,000 ms, time of echo (TE) = 30 ms, field of view (FOV) = 220 × 220 mm^2^, slice thickness = 2.2 mm, slice gap = 0.4 mm, flip angle = 70 ^°^, voxel size = 2.0 × 2.0 × 2.2 mm^3^, slice number = 52, volumes = 400.

### Resting-state fMRI preprocessing

Data Processing Assistant for Resting-State fMRI Analysis Toolkit (DPARSFA, V5.2) (Yan and Zang, 2010) was used to preprocess the rs-fMRI data. (1) Converting the DICOM images into the NIFTI format, (2) deleting the first 10 time points, (3) slice-timing, (4) realignment (participants with linear shifting distances more than 2.5 mm or rotation angles more than 2.5° were excluded), (5) spatially normalizing the fMRI figures to the Montreal Neurological Institute (MNI) space and resampling to 3 × 3 × 3 mm^3^ resolution, (6) detrending, (7) regressing several spurious variances, including 24 head motion parameters (Satterthwaite et al., 2012), cerebrospinal fluid signals, and white matter signals, (8)band-pass temporal filtering between 0.01–0.08 Hz, and (9) framewise displacement (FD) (Power et al., 2012)was calculated for each time point, and participants with mean FD exceeding 0.5 mm were excluded.

### sReHo and dReHo analysis

Kendall’s coefficient of concordance (KCC) was utilized to calculate the sReHo and dReHo of a voxel’s time series and its adjacent voxels (Zang et al., 2004). In our study, the cluster size of KCC was set to be 27, which is adequate for covering all directions in 3D space and to optimize the trade-off between mitigation of partial volume effects and generation of Gaussian random fields (Jiang and Zuo, 2016). However, considering that the size of cluster to be measured might affect KCC value, we validated the results using additional two sorts of cluster size (7 and 19 voxels, respectively). For sReHo, the KCC value of a given voxel with those of its nearest neighbors was calculated through DPARSFA. Then, the sReHo map of each subject was obtained and transformed into standardized *z*-score. The dReHo analysis was performed using temporal dynamic analysis toolkits (Yan et al., 2017). A method based on sliding window was used to describe the temporal dynamic patterns. It is rather remarkable that the window length is a key parameter. According to prior studies, the minimum window length should exceed 1/*f_min_*, where *f_min_* denoted the minimum frequency of time courses (Leonardi and Van De Ville, 2015). Therefore, a window size of 100 TRs (100 s) and a window overlap of 60% (step size by 40 TRs, 40s) were selected (Wen et al., 2021). We also tested the results of other window sizes and overlaps, and the specific information is described in the validation analysis. The standard deviation of the dReHo was calculated to estimate temporal variability. Then, the time variability map of each subject was normalized into a *z*-score matrix. At last, spatial smoothing with a 6 mm full-width at half-maximum (FWHM) Gaussian kernel was performed for the sReHo and dReHo maps.

### Statistical analysis

Demographic and clinical characteristics of participants were analyzed using IBM SPSS22.0 software. The differences in age, gender and mean FD among mTLE-IPI, mTLE-NO and HC were, respectively, analyzed with one-way analysis of variance (ANOVA), Chi-square test and Kruskal–Wallis test (*p* < 0.05). Difference in lateralization between mTLE-IPI and mTLE-NO was analyzed with Chi-square test (*p* < 0.05), while differences in epilepsy duration, NHS3 score and the number of ASMs were analyzed with Mann–Whitney U test, respectively, (*p* < 0.05).

Whole-brain voxel-wise comparisons of sReHo and dReHo among the three groups were employed by one-way ANOVA using SPM12 toolkit, with age, gender and mean FD as covariates. A gaussian random field (GRF) correction was conducted for the *F*-value map (voxel-wise *p* < 0.005 and cluster-level *p* < 0.05, cluster extent threshold *k* > 30). Then, a new mask (the brain regions where the *F* value changed significantly) was applied to perform the secondary analyses through two-sample *t*-test (GRF corrected, *p*_voxel_ *<* 0.005, *p*_cluster_ *<* 0.05, *k* > 30) between mTLE-IPI and HC, mTLE-NO and HC, mTLE-IPI and mTLE-NO.

### Correlation analysis

To identify whether the sReHo and dReHo abnormalities were associated with clinical characteristics, we conducted Spearman correlation analyses of the sReHo and dReHo values extracted from brain clusters showing significant differences on F map with epilepsy duration, NHS3 score and the number of ASMs, respectively. *p* < 0.05 was set for the statistically significant threshold.

### Validation analyses

First, the ReHo’s proposer (Zang et al., 2004) claimed that the size of cluster (7, 19, or 27 voxels) to be measured had a considerable effect on KCC; that is, the results may differ depending on this number. Although the generally accepted cluster size is 27 voxels, we also tested additional two sorts of cluster size (7 and 19 voxels, respectively) to investigate how they would affect the outcomes. Second, since the optimal window size and step size of the sliding window method have not yet been identified, different window sizes/step sizes (100/20 TRs and 80/30 TRs) were examined to verify the reproducibility of dReHo results.

## Results

### Demographic and clinical information

The demographic and clinical information of the three groups are summarized in Table 1. No significant differences were detected in terms of age, gender or mean FD among three groups. For the comparison between mTLE-IPI and mTLE-NO, there were no notable differences between the two groups in lateralization, epilepsy duration, NHS3 score or the number of ASMs.

**Table 1:** Demographic and clinical information of participants.

Characteristics	mTLE-IPI	mTLE-NO	HC	Statistic	*p* value
Age (year)	29.92 ± 8.05	29.54 ± 8.76	29.52 ± 8.68	*F* = 0.019	0.981
Gender (male/female)	14/11	14/21	15/18	χ^2^ = 1.541	0.469
Mean FD (mm)	0.07 (0.05)	0.05 (0.06)	0.05 (0.05)	*F* = 2.038	0.361
Lateralization (left/right)	10/15	17/18	–	χ^2^ = 0.433	0.511
Epilepsy duration (year)	11.00 (18.00)	8.00 (10.00)	–	Z = −0.661	0.509
NHS3 score	11.00 (7.00)	10.00 (7.00)	–	Z = −0.346	0.729
The number of ASMs	2.00 (1.00)	3.00 (1.00)	–	Z = −0.703	0.482

mTLE-IPI, mTLE patients with initial precipitating injury; mTLE-NO, mTLE without IPI; HC, healthy controls; FD, framewise displacement; NHS3, national hospital seizure severity scale; ASMs, antiseizure medications. Values are mean ± standard deviation; others are median (interquartile range).

### Group differences in sReHo

**Figure 1:**
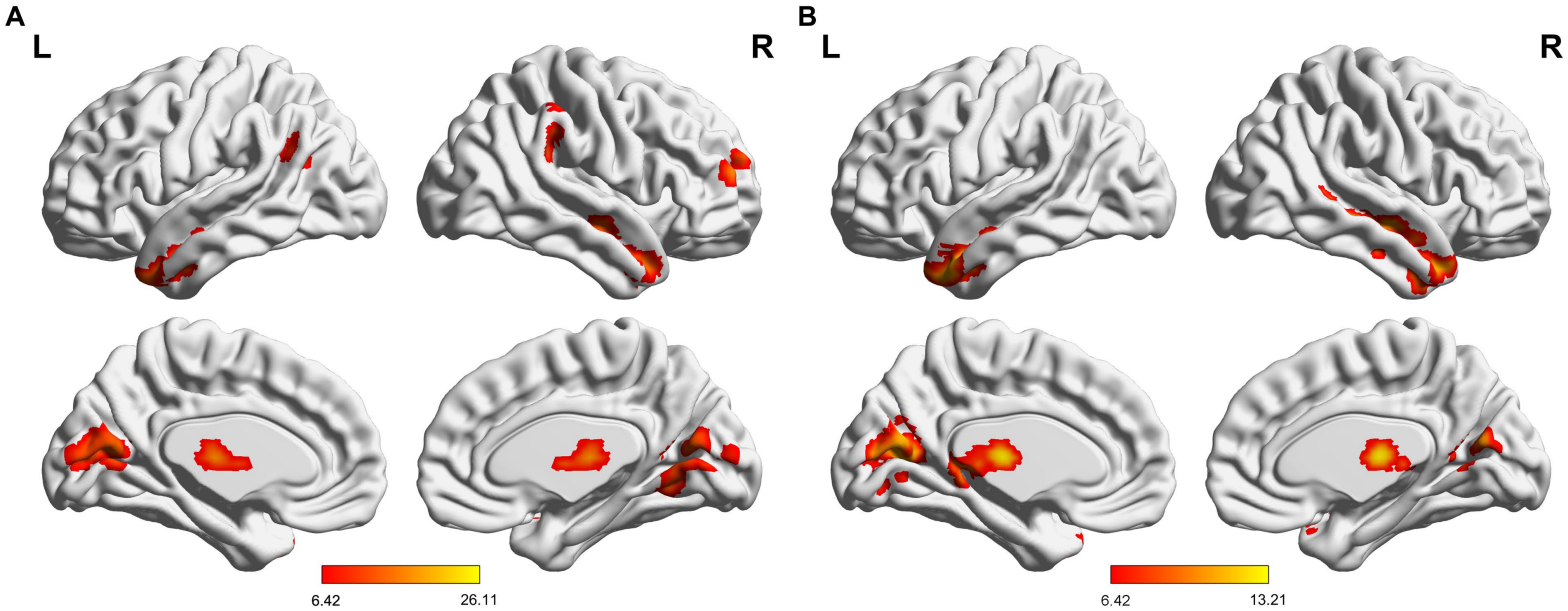
**(A)** Significant alterations of sReHo among three groups; **(B)** Significant alterations of dReHo among three groups. sReHo, static regional homogeneity; dReHo, dynamic regional homogeneity; L, left; R, right.

**Figure 2:**
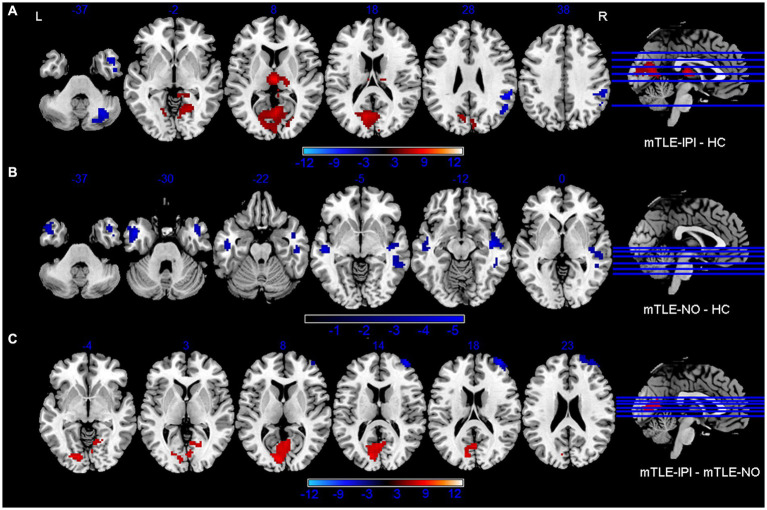
Brain regions with significant alterations of sReHo between mTLE-IPI and HC **(A)**, mTLE-NO and HC **(B)**, mTLE-IPI and mTLE-NO **(C)**. GRF corrected; voxel-wise *p* < 0.005, cluster-level *p* < 0.05. Warm colors indicate increased sReHo, while cold colors indicated decreased sReHo. sReHo, static regional homogeneity; mTLE-IPI, mTLE patients with initial precipitating injury; mTLE-NO, mTLE patients without initial precipitating injury; HC, healthy controls; GRF, Gaussian random field theory; L, left; R, right.

The results revealed that mTLE-IPI and mTLE-NO had distinct patterns of sReHo alterations (Figure 1 and Table 2). Compared with HC, mTLE-IPI showed decreased sReHo in the right inferior temporal gyrus (ITG), middle temporal gyrus (MTG), cerebellum crus1/ crus2, inferior parietal lobule (IPL, including angular gyrus and supramarginal gyrus), while increased sReHo in the bilateral thalamus, lingual gyrus (LG), calcarine (CAL) and cuneus. mTLE-NO showed decreased sReHo in the bilateral MTG, left ITG and right superior temporal gyrus (STG). Compared with mTLE-NO, mTLE-IPI manifested decreased sReHo in the right dorsolateral prefrontal cortex (DLPFC), while increased sReHo in the bilateral CAL and right LG (Figure 2).

**Table 2:** Brain regions with changed sReHo and dReHo among the three groups.

Indices	Cluster	Voxels	Brain region	Peak intensity	MNI coordinate
sReHo	1	68	Cerebellum_Crus1 _R	13.230	30, −66, −39
			Cerebellum_Crus2 _R	12.160	33, −67, −40
	2	114	Temporal_Inf_L	16.302	−45, 6, −33
			Temporal_Mid_L	6.981	−51, −4, −25
	3	118	Temporal_Sup_R	17.006	45, −12, −12
			Temporal_Inf_R	11.000	45, 8, −34
			Temporal_Mid_R	9.151	48, 8, −29
	4	326	Calcarine_R	13.083	3, −66, 12
			Calcarine_L	10.160	−2, −67, 14
			Lingual_R	9.773	1, −68, 9
			Lingual_L	6.677	−12, −70, 4
			Cuneus_L	9.644	−10, −72, 22
			Cuneus_R	7.172	6, −75, 20
	5	64	Thalamus_R	21.106	3, −16, 11
			Thalamus_L	6.571	5, −6, 5
	6	72	DLPFC_R	16.239	39, 57, 18
	7	54	Angular_R	9.108	48, −43, 28
			SupraMarginal_R	10.635	57, −39, 27
dReHo	1	233	Temporal_Pole_Mid_R	12.152	42, 9, −36
			Temporal_Mid_R	7.267	47, 4, −28
			Temporal_Inf_R	7.592	45, 1, −33
			Temporal_Sup_R	7.821	45, −15,-9
	2	151	Temporal_Mid_L	12.488	−57, 9, −27
			Temporal_Inf_L	9.127	−46, 4, −34
	3	311	Calcarine_L	13.210	−9, −69, 21
			Calcarine_R	6.314	2, −70, 19
			Cuneus_L	9.356	−9, −73, 27
			Lingual_R	5.661	13, −65, −3
			Lingual_L	5.756	−9, −70, −1
	4	131	Thalamus_L	10.140	−5, −16, 7

GRF corrected, voxel-wise *p* < 0.005, cluster-level *p* < 0.05; sReHo, static regional homogeneity; dReHo, dynamic regional homogeneity; DLPFC, dorsolateral prefrontal cortex; L, left; R, right.

### Group differences in dReHo

**Figure 3:**
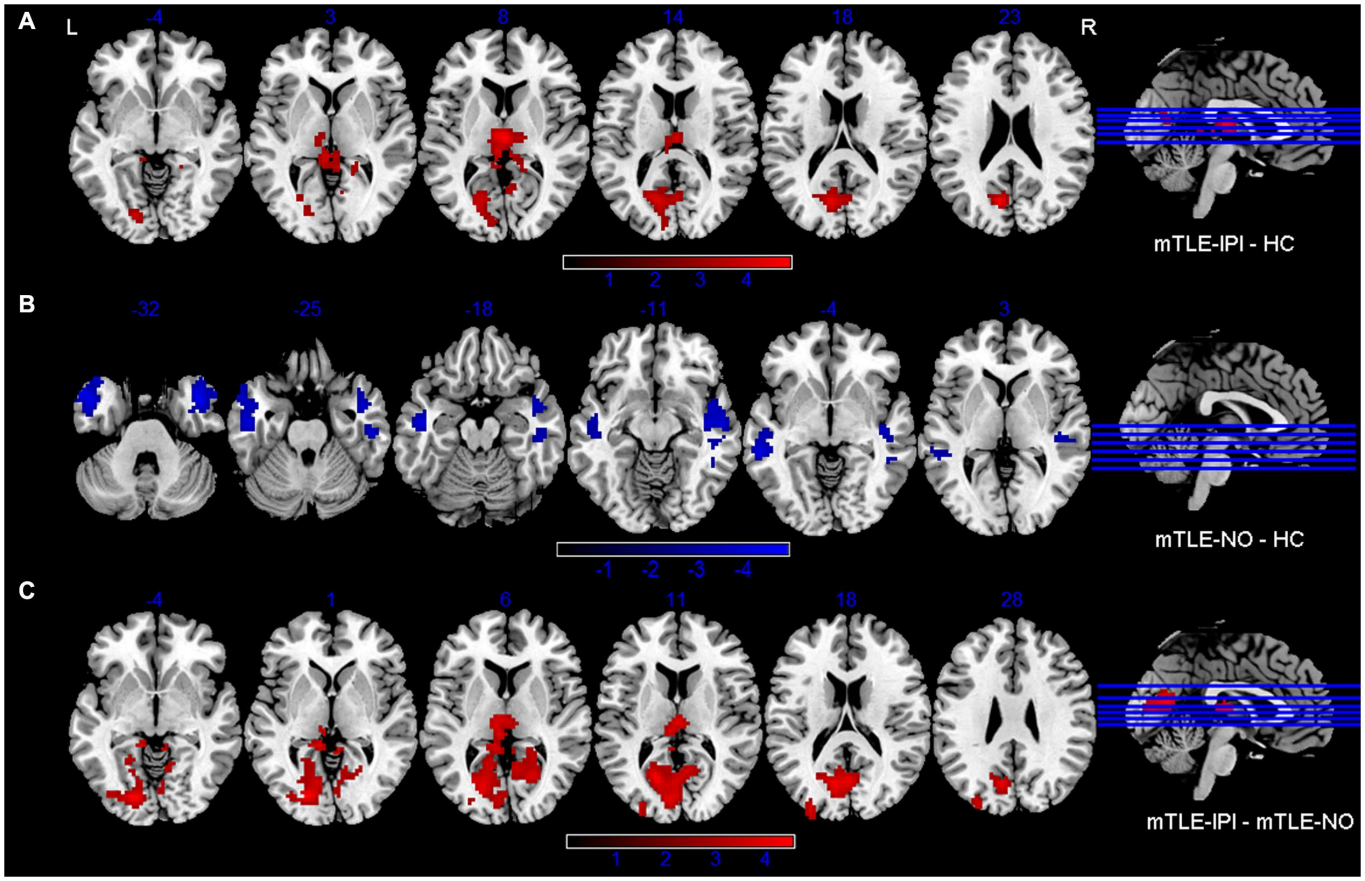
Brain regions with significant alterations of variability of dReHo between mTLE-IPI and HC **(A)**, mTLE-NO and HC **(B)**, mTLE-IPI and mTLE-NO **(C)**. GRF corrected; voxel-wise *p* < 0.005, cluster-level *p* < 0.05. Warm colors indicate increased variability of dReHo, while cold colors indicated decreased variability of dReHo. dReHo, dynamic regional homogeneity; mTLE-IPI, mTLE patients with initial precipitating injury; mTLE-NO, mTLE patients without initial precipitating injury; HC, healthy controls; GRF, Gaussian random field theory; L, left; R, right.

The three groups presented significantly different variability of dReHo (Figure 1 and Table 2). Compared with HC, mTLE-IPI showed increased variability of dReHo in the bilateral thalamus, left CAL and cuneus. mTLE-NO, on the other hand, showed decreased variability of dReHo in the bilateral MTG, ITG and right STG. Compared with mTLE-NO, mTLE-IPI manifested increased variability of dReHo in the bilateral CAL, LG, left cuneus and thalamus (Figure 3).

### Correlation analysis

**Figure 4:**
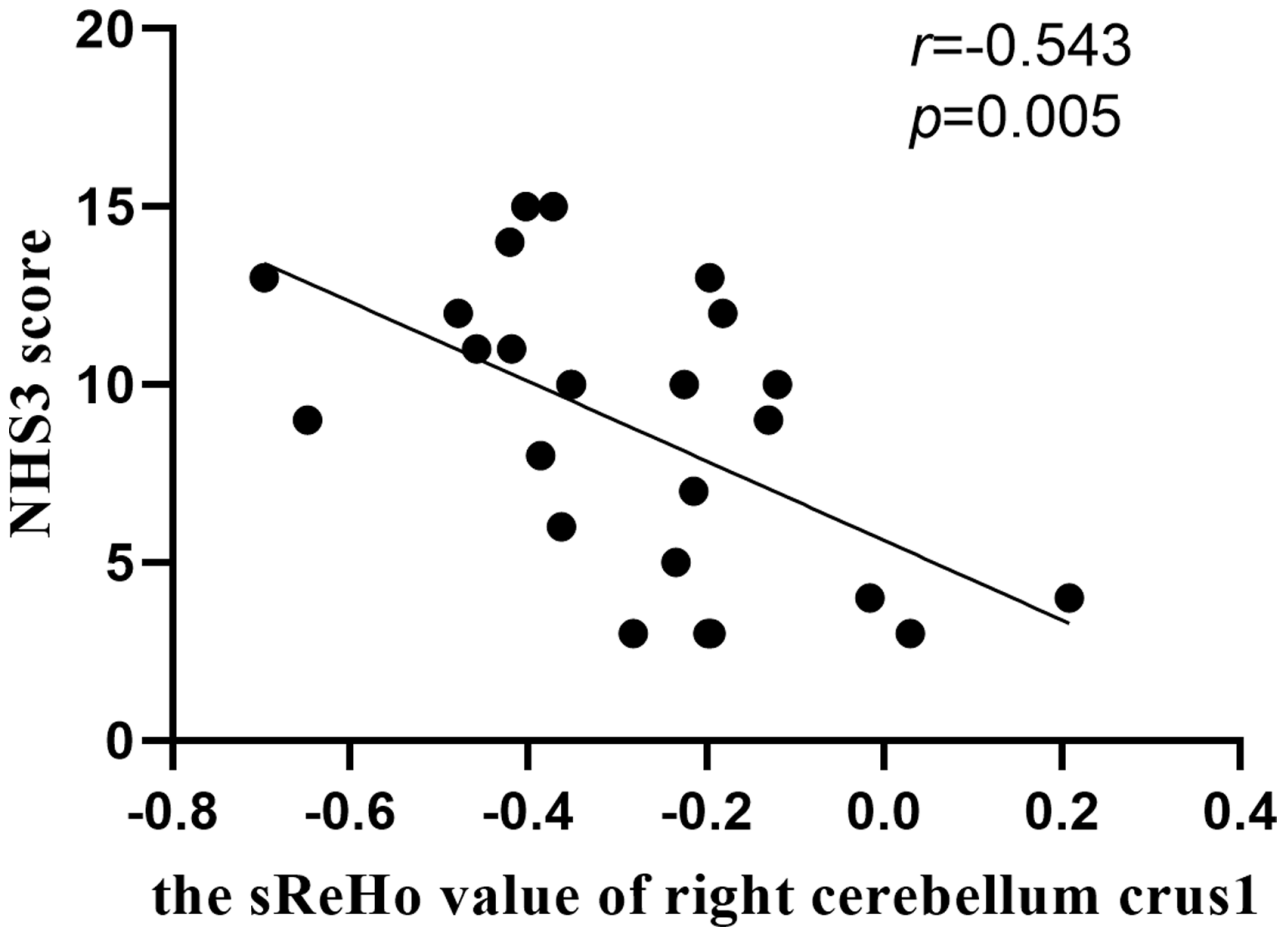
The correlation between the sReHo value of right cerebellum crus1 and NHS3 score in patients with mTLE-IPI.

As shown in Figure 4, the NHS3 score was negatively correlated with sReHo in right cerebellum crus1 in mTLE-IPI patients (*r* = −0.543, *p* = 0.005). However, there was no discernible correlation between the abnormal variability of dReHo and epilepsy duration, NHS3 score or the number of ASMs.

### Validation analyses

Two different cluster sizes (7 and 19 voxels, respectively) were investigated to find out how they might impact the results. The new results were mostly consistent with the results reported above. However, there were slight variances in the outcomes: (1) when 7 and 19 voxels were selected, respectively, to calculate sReHo, the difference in sReHo between mTLE-IPI and mTLE-NO patients in the right cerebellar Crus1/Crus2 vanished, (2) when 7 voxels were selected, the right inferior parietal lobule and right temporal lobe of the mTLE-IPI lost their decreased sReHo as compared to HC, and (3) the right dorsolateral prefrontal cortex’s decreased sReHo and the left thalamus’s increased variability of dReHo of mTLE-IPI vanished when compared to mTLE-NO. For more information, please consult Supplementary Figures S1–S4. What’s more, different window sizes/step sizes (100/20 TRs and 80/30 TRs) were used to validate the variability of dReHo. The new results were basically consistent with the main results. Please see Supplementary Figures S5, S6 for details.

## Discussion

Despite the fact that there have been numerous research on static and dynamic ReHo in the past, we found that the majority of them highlighted the differences between all patients and healthy controls, without detailed grouping of patients, such as with or without IPI, which may lead to inconsistent results. Therefore, we explored the changes in spontaneous neural activity in the three groups from the perspectives of sReHo and dReHo to investigate if IPI had an effect on cognitive impairment in mTLE patients. As results showed, the differences between the three groups were mainly located in the bilateral thalamus, occipital lobe and temporal lobe neocortex, and the activation brain regions of sReHo and dReHo were basically the same, suggesting that the dysfunction of these brain regions was reflected not only in the static state, but also in the dynamic state (Duma et al., 2022, 2023). Besides, mTLE-IPI had decreased sReHo in right cerebellum, IPL and DLPFC compared with HC and mTLE-NO respectively, implying a complementarity between sReHo and dReHo.

The thalamus is a key relay station for communicating information between the cortex and subcortical structures, as well as a crucial extratemporal structure for regulating and propagating TLE epileptogenic discharges (Keller et al., 2014). It functions critically in mental activity, the arousal system of the brain, emotion and movement (Ward, 2013). A significant and expanding body of literature supports the functional and structural impairment of the thalamus in patients with mTLE (Barron et al., 2015; Chen et al., 2015; Gleichgerrcht et al., 2021). In a mouse model of temporal lobe epilepsy (Fei et al., 2022), hippocampal subicular pyramidal neurons were demonstrated to project to the anterior nucleus of the thalamus. As we know, hippocampal neurons are easily damaged by IPI (Janz et al., 2018). Due to the abnormal propagation of hippocampus discharges, we have reason to believe that not only were local neurons in the bilateral thalamus over-activated, but also the stability of neuronal activity was disrupted in mTLE-IPI patients. The abnormal spontaneous brain activity in the thalamus may be associated with attention deficiency, memory impairment and emotion change in mTLE-IPI patients. The dReHo variability of the left thalamus in mTLE-IPI patients was higher than that in mTLE-NO patients, suggesting that mTLE-IPI patients may have severer impairment of thalamic regional brain activity than mTLE-NO patients.

In contrast to HC and mTLE-NO patients, our investigation demonstrated that abnormal sReHo or dReHo took place in the bilateral visual cortex of mTLE-IPI patients. The visual network, which is located in occipital lobe and includes CAL, LG, cuneus and so on, is the key part of the brain for visual information integration and attention processing (Bezdek et al., 2015). A section of the primary visual cortex known as the CAL receives visual information directly from visual stimuli (Wang et al., 2022). LG is involved in the processing of visual memory, especially in the processing of words. Integrating visual information is believed to be a crucial function of the cuneus (Yang et al., 2022). Numerous investigations (Pang et al., 2021; Wills et al., 2021) have revealed that mTLE patients have functionally impaired occipital cortex. In line with these studies, our results reflect that patients with mTLE-IPI may be more likely to have impaired visual networks compared with mTLE-NO and HC. Inflammatory mediators, which can be produced by febrile convulsion as well as intracranial infection, will raise glutamatergic neurons’ excitability (Rana and Musto, 2018; Mosili et al., 2020). The high density of glutamate receptors in the occipital cortex may lead the neurons to be in a more excited state and have a long-term influence on the neurons as the disease advances, resulting in unstable local neuronal activity in mTLE-IPI patients. The changed sReHo and dReHo in the bilateral visual cortex support the decline in patients’ visual memory, spatial attention and ability to recognize colors and letters (Song et al., 2022). We noted that there were subtle differences in the areas of spontaneous brain activity alterations in the visual cortex. One possible explanation is that the combination of static and dynamic ReHo is more sensitive in reflecting brain abnormalities.

Our findings also suggested that both sReHo and the variability of dReHo in the bilateral MTG, left ITG and right STG in mTLE-NO patients were lower than in HC. Besides, the sReHo in right MTG and ITG was decreased in mTLE-IPI patients. These abnormal ReHo changes could be a result of the temporal neocortex’s abnormal neuroplasticity being influenced by frequent abnormal discharges (Jiang et al., 2021). The MTG (Binder et al., 2009) and ITG (Ojemann et al., 2002) play various roles in memory, auditory processing and emotion, while the STG houses a part of the primary and association auditory cortices (Song et al., 2022). Although results varied slightly due to the methodological differences, we could infer that mTLE patients (no matter with or without IPI) may be more prone to have functional deficits in social cognition, auditory processing and emotion management. We have to mention that a task state functional magnetic resonance imaging study (Trimmel et al., 2018) found that the right TIG was activated during picture naming in mTLE patients. Our findings, however, showed a reduction in sReHo in the right temporal neocortex. This inconsistency could be attributed to the fact that the patients in current study did not perform tasks such as picture naming during the scan.

The cerebellum not only plays an important role in the motor dysfunction of mTLE, but also participates in the impairment of cognitive function (Argyropoulos et al., 2020; Hao et al., 2022). The posterior cerebellum, such as crus 1/crus 2, corresponds to the prefrontal cortex (Wang et al., 2021) and IPL (Zhou et al., 2019) structurally and functionally, and participates in advanced cognitive function, especially executive control function. The abnormal sReHo detected in right cerebellum crus1/ crus2 and IPL may imply the disruption of neuronal activity in cerebellum-related neural circuits, supporting the higher-level cognitive and executive dysfunction in mTLE-IPI patients. Additionally, our results indicated that the NHS3 score was negatively correlated with sReHo in right cerebellum crus1 in mTLE-IPI, suggesting that the lower the consistency of local neuronal activity in the right cerebellum crus1, the poorer the patient’s condition.

Interestingly, sReHo in the right DLPFC of mTLE-IPI patients was decreased when compared with mTLE-NO patients, whereas there was no significant difference when compared with HC. Therefore, we speculated that one of the two groups had a slight increase in sReHo of the right DPLFC, while the other had a tiny decrease. Increases or decreases in sReHo, particularly in the DPLFC, which is widely considered to be a vital cortical region involved in cognitive function (Qin et al., 2020), may cause neuronal activity to deviate from the normal range, which is detrimental to normal cognitive activity.

It is noteworthy that the number of brain regions with significant differences between the three groups changed slightly when we varied the cluster size, and the smaller the cluster size was the fewer distinct brain regions there were. This is consistent with the assertion (Zang et al., 2004) that “larger size of cluster yielded more differences.” So, we speculated smaller cluster size probably decreased the detection of spontaneous brain activity and hindered ReHo temporal variability. Recent years, several researchers (Shinn et al., 2023) have advocated using the parcellation method to determine the ReHo value of each parcellated brain region, such that at least one could have a region specific value of homogeneity. It is worth investigating which method can better characterize the constancy of local neural activity.

## Limitations

There are several limitations in this study. To mention first, a larger sample size is needed to improve the credibility of our findings. The sample size in this study is relatively small. More individuals should be included in follow-up studies. Secondly, for mTLE-IPI patients, we did not further subdivide them. There may be different patterns of intrinsic brain activities among mTLE patients caused by different IPI. Thus, more detailed grouping is required to deepen the understanding of neuropathological changes of different mTLE-IPI subtypes, which is of profound significance for guiding clinical treatment. Thirdly, the effect of ASMs cannot be ignored. ASMs may confound the results of this study since they can inhibit the epileptogenesis by preventing the excitatory transmission of neurons (Miziak et al., 2020), which may have a certain impact on regional brain activities. Although we compared the number of ASMs between the two groups, it may be far from enough. Finally, the current study is cross-sectional. In order to better explain the mechanism of IPI on mTLE and its ongoing effect, longitudinal research is necessary.

## Conclusion

The current study found that there were different patterns of local neuronal activity alterations in mTLE-IPI and mTLE-NO, and the severity of abnormalities in mTLE-IPI patients was greater than that in mTLE-NO patients, especially in the bilateral thalamus and visual cortex. Besides, there were abnormal sReHo in the right temporal neocortex no matter in mTLE-IPI or mTLE-NO patients. Furthermore, our study also suggested that combining the static and dynamic indicators could build better models of brain function and dysfunction. In a word, these findings can help us comprehend how IPI affects the impaired brain activity of mTLE patients and can assist in carrying out timely intervention for patients with mTLE-IPI in order to control the progress of the disease effectively.

## Data availability statement

The raw data supporting the conclusions of this article will be made available by the authors, without undue reservation.

## Ethics statement

The studies involving human participants were reviewed and approved by Research Ethical Committee of The First Affiliated Hospital of Zhengzhou University. Written informed consent to participate in this study was provided by the participants’ legal guardian/next of kin.

## Author contributions

XM wrote the first draft of the manuscript. XM and XZ contributed to design of the study and performed data and statistical analysis. XM, XZ, CS, KM, and KW collected the data. YL provided the clinical diagnosis of the participants. XW and SH provided the methodological advice. XZ, CS, and SH proofread the manuscript. YaZ, YoZ, and JC supervised the conduct of the study. All authors contributed to the article and approved the submitted version.

## Conflict of interest

The authors declare that the research was conducted in the absence of any commercial or financial relationships that could be construed as a potential conflict of interest.

## Publisher’s note

All claims expressed in this article are solely those of the authors and do not necessarily represent those of their affiliated organizations, or those of the publisher, the editors and the reviewers. Any product that may be evaluated in this article, or claim that may be made by its manufacturer, is not guaranteed or endorsed by the publisher.
