# Supplementary material for: Alterations in static and dynamic regional homogeneity in mesial temporal lobe epilepsy with and without initial precipitating injury
Source: Front Neurosci. 2023 Aug 1;17:1226077. doi: 10.3389/fnins.2023.1226077 (PMC10434245; doi:10.3389/fnins.2023.1226077)
Supplement: Supplementary file 1 [file Data_Sheet_1.docx]

Supplementary Material

**Alterations in Static and Dynamic ReHo in Mesial Temporal Lobe Epilepsy with and without Initial Precipitating Injury**

Xinyue Mao^1,2,3,4,5,6,7†^, Xiaonan Zhang^1,2,3,4,5,6,7†^, Chengru Song^1,2,3,4,5,6,7^, Keran Ma^1,2,3,4,5,6,7^, Kefan Wang^1,2,3,4,5,6,7^, Xin Wang^1,2,3,4,5,6,7^, Yajun Lian^8^, Yong Zhang^1,2,3,4,5,6,7^, Shaoqiang Han^1,2,3,4,5,6,7*^, Jingliang Cheng^1,2,3,4,5,6,7*^ and Yan Zhang^1,2,3,4,5,6,7*^

^1^Department of Magnetic Resonance Imaging, The First Affiliated Hospital of Zhengzhou University, Zhengzhou, China, ^2^ Key Laboratory for Functional Magnetic Resonance Imaging and Molecular Imaging of Henan Province, Zhengzhou, China, ^3^Engineering Technology Research Center for Detection and Application of Brain Function of Henan Province, Zhengzhou, China, ^4^ Engineering Research Center of Medical Imaging Intelligent Diagnosis and Treatment of Henan Province, Zhengzhou, China, ^5^Key Laboratory of Magnetic Resonance and Brain Function of Henan Province, Zhengzhou, China, ^6^Key Laboratory of Brain Function and Cognitive Magnetic Resonance Imaging of Zhengzhou, Zhengzhou, China, ^7^Key Laboratory of Imaging Intelligence Research Medicine of Henan Province, Zhengzhou, China, ^8^Department of Neurology, The First Affiliated Hospital of Zhengzhou University, Zhengzhou, China

^†^These authors have contributed equally to this work and share first authorship

*Correspondence:

Yan Zhang: [fcczhangy61@zzu.edu.cn](mailto:fcczhangy61@zzu.edu.cn)

Shaoqiang Han: [shaoqianghan@163.com](mailto:shaoqianghan@163.com)

Jingliang Cheng: [fccchengjl@zzu.edu.cn](mailto:fccchengjl@zzu.edu.cn)

## 1. Supplementary Figure 1


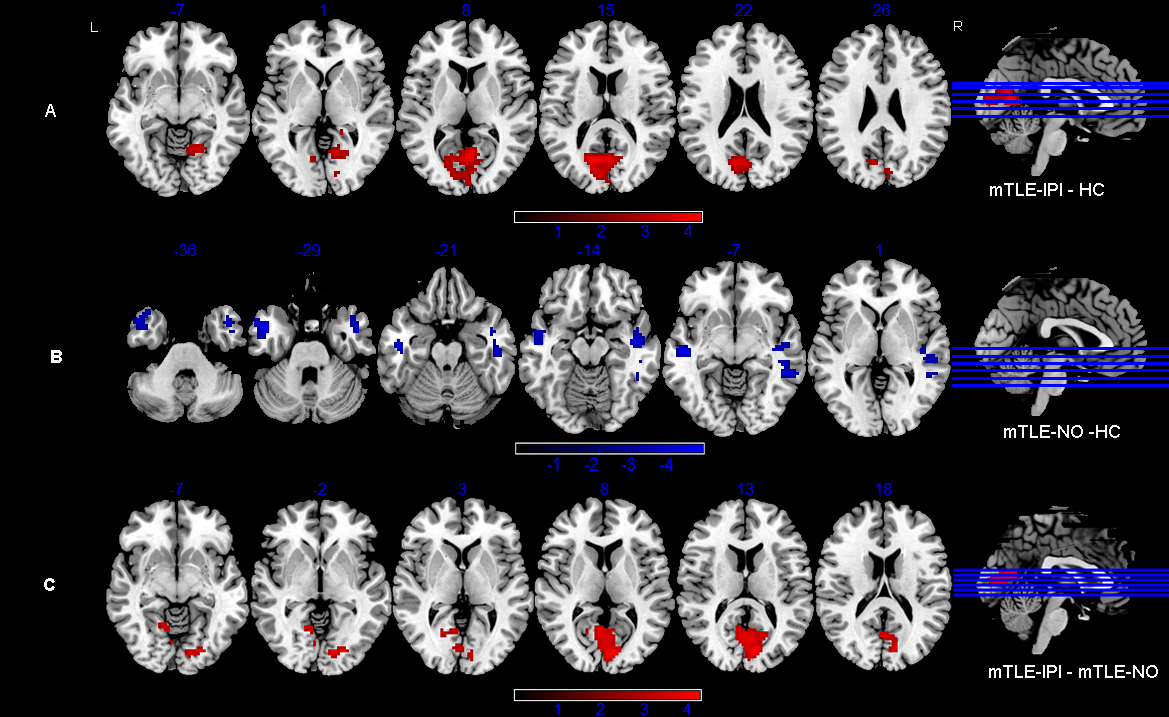


**Supplementary Figure 1** When 7 voxels were selected to calculate sReHo, the results were as follows. Brain regions showed significant alterations in sReHo between mTLE-IPI and HC **(A)**, mTLE-NO and HC **(B)**, mTLE-IPI and mTLE-NO **(C)**. GRF corrected; voxel-wise *p* < 0.005, cluster-level *p* < 0.05. Warm colors indicate increased sReHo, while cold colors indicated decreased sReHo. mTLE-IPI, mTLE patients with initial precipitating injury; mTLE-NO, mTLE patients without initial precipitating injury; HC, healthy controls; GRF, Gaussian random field theory; L, left; R, right.

## 2. Supplementary Figure 2


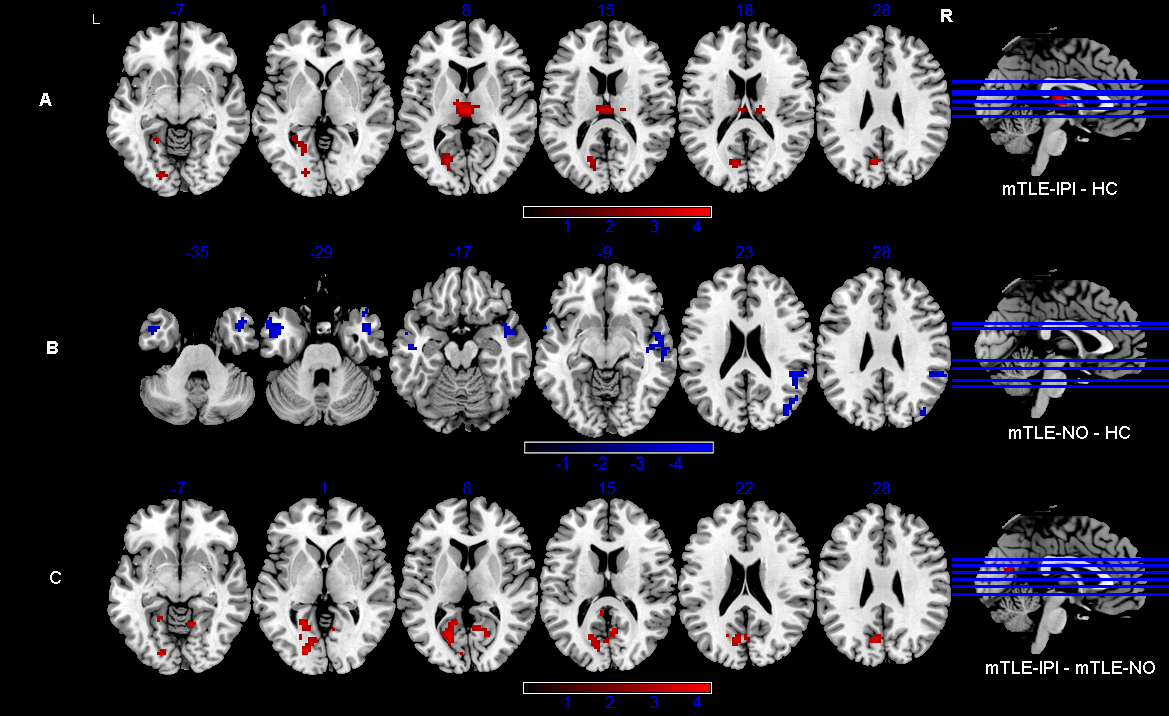


**Supplementary Figure 2** When 7 voxels were selected to calculate dReHo, the results were as follows. Brain regions showed significant alterations in dReHo between mTLE-IPI and HC **(A)**, mTLE-NO and HC **(B)**, mTLE-IPI and mTLE-NO **(C)**. GRF corrected; voxel-wise *p* < 0.005, cluster-level *p* < 0.05. Warm colors indicate increased dReHo, while cold colors indicated decreased dReHo. mTLE-IPI, mTLE patients with initial precipitating injury; mTLE-NO, mTLE patients without initial precipitating injury; HC, healthy controls; GRF, Gaussian random field theory; L, left; R, right.

**3. Supplementary Figure 3**


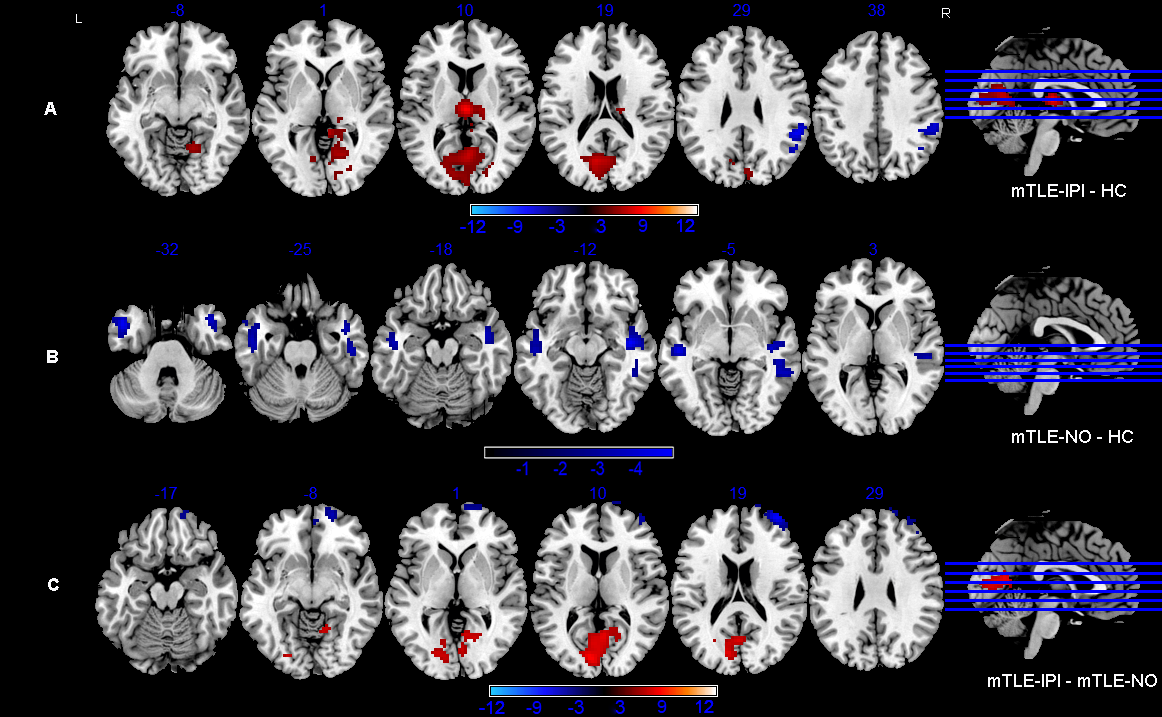


**Supplementary Figure 3** When 19 voxels were selected to calculate sReHo, the results were as follows. Brain regions showed significant alterations in sReHo between mTLE-IPI and HC **(A)**, mTLE-NO and HC **(B)**, mTLE-IPI and mTLE-NO **(C)**. GRF corrected; voxel-wise *p* < 0.005, cluster-level *p* < 0.05. Warm colors indicate increased sReHo, while cold colors indicated decreased sReHo. mTLE-IPI, mTLE patients with initial precipitating injury; mTLE-NO, mTLE patients without initial precipitating injury; HC, healthy controls; GRF, Gaussian random field theory; L, left; R, right.

**4. Supplementary Figure 4**


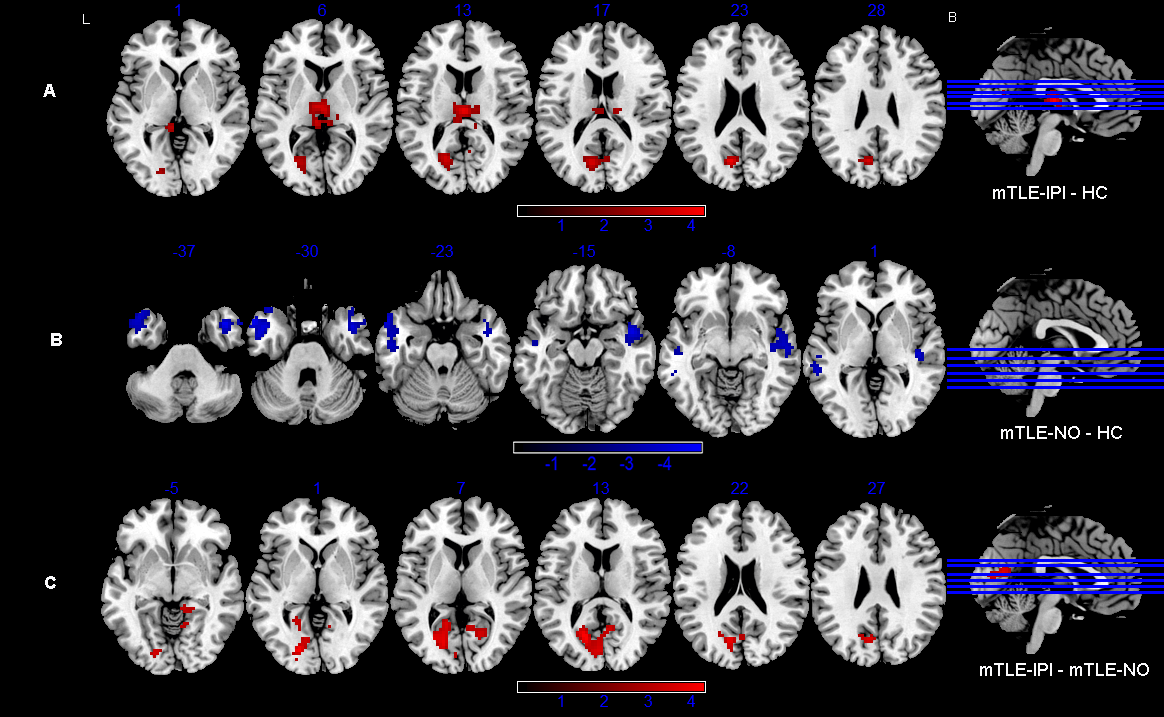


**Supplementary Figure 4** When 19 voxels were selected to calculate dReHo, the results were as follows. Brain regions showed significant alterations in dReHo between mTLE-IPI and HC **(A)**, mTLE-NO and HC **(B)**, mTLE-IPI and mTLE-NO **(C)**. GRF corrected; voxel-wise *p* < 0.005, cluster-level *p* < 0.05. Warm colors indicate increased dReHo, while cold colors indicated decreased dReHo. mTLE-IPI, mTLE patients with initial precipitating injury; mTLE-NO, mTLE patients without initial precipitating injury; HC, healthy controls; GRF, Gaussian random field theory; L, left; R, right.

**5. Supplementary Figure 5**


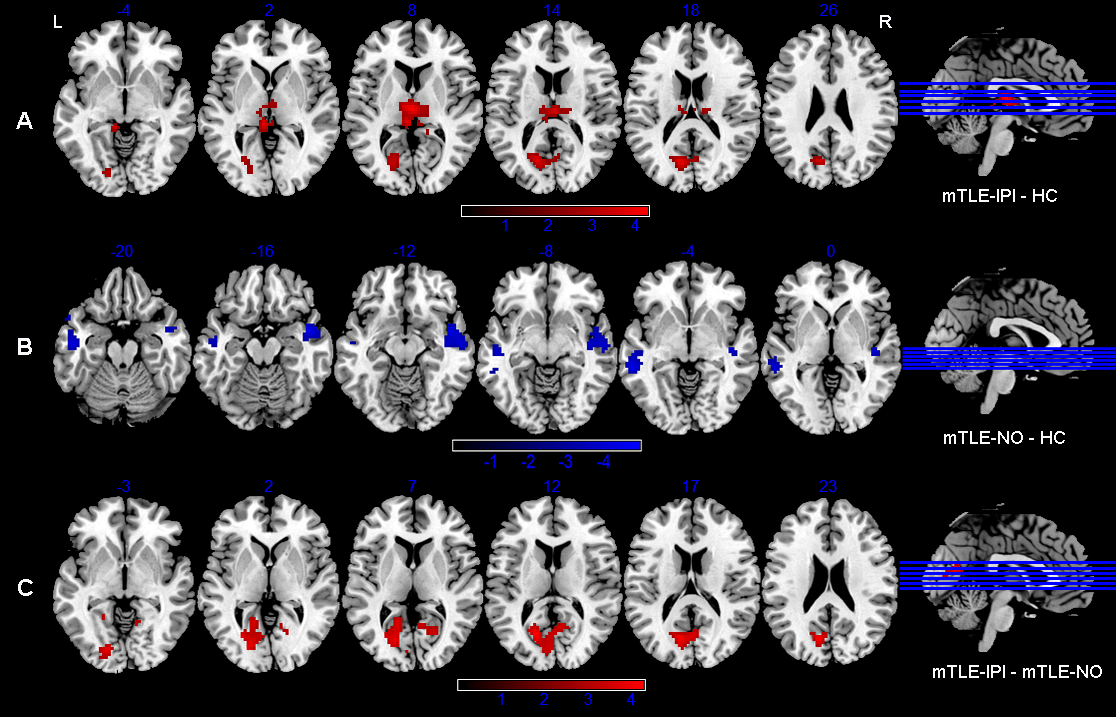


**Supplementary Figure 5** When the window size was 100TRs and the step size was 20TRs, the results were as above. Brain regions showed significant alterations of variability of dReHo between mTLE-IPI and HC **(A)**, mTLE-NO and HC **(B)**, mTLE-IPI and mTLE-NO **(C)**. GRF corrected; voxel-wise *p* < 0.005, cluster-level *p* < 0.05. Warm colors indicate increased variability of dReHo, while cold colors indicated decreased variability of dReHo. dReHo, dynamic regional homogeneity; mTLE-IPI, mTLE patients with initial precipitating injury; mTLE-NO, mTLE patients without initial precipitating injury; HC, healthy controls; GRF, Gaussian random field theory; L, left; R, right.

## 6. Supplementary Figure 6


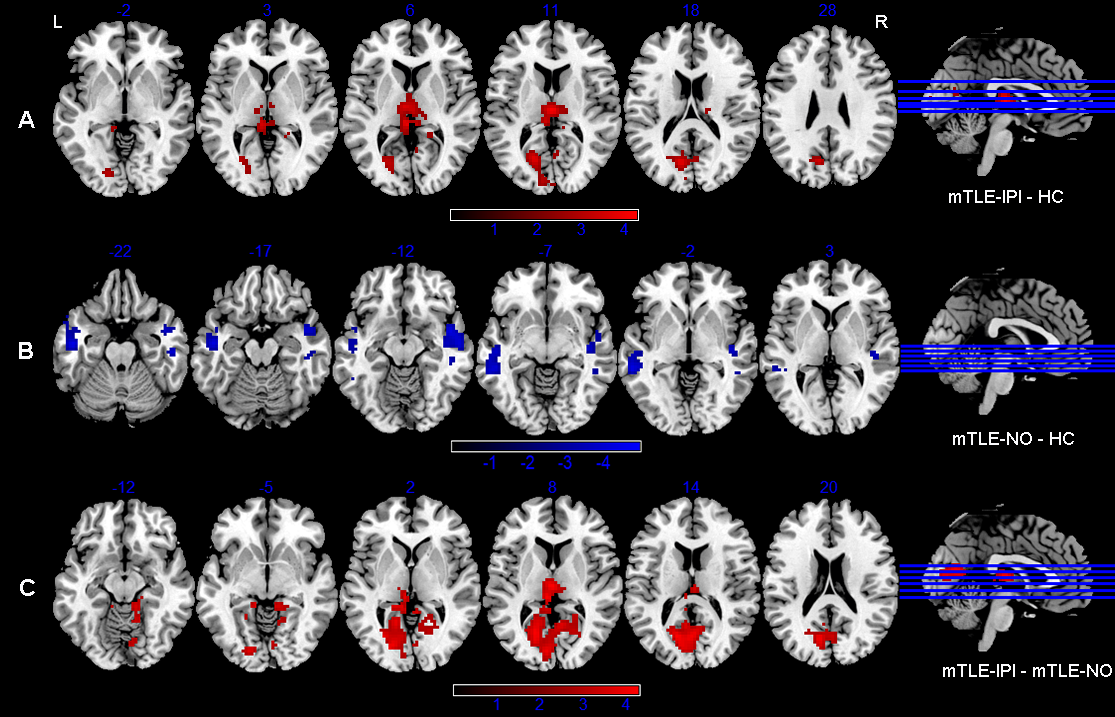


**Supplementary Figure 6** When the window size was 80TRs and the step size was 30TRs, the results were as above. Brain regions showed significant alterations of variability of dReHo between mTLE-IPI and HC **(A)**, mTLE-NO and HC **(B)**, mTLE-IPI and mTLE-NO **(C)**. GRF corrected; voxel-wise *p* < 0.005, cluster-level *p* < 0.05. Warm colors indicate increased variability of dReHo, while cold colors indicated decreased variability of dReHo. dReHo, dynamic regional homogeneity; mTLE-IPI, mTLE patients with initial precipitating injury; mTLE-NO, mTLE patients without initial precipitating injury; HC, healthy controls; GRF, Gaussian random field theory; L, left; R, right.
